# Zinc and copper toxicity in host defense against pathogens: *Mycobacterium tuberculosis* as a model example of an emerging paradigm

**DOI:** 10.3389/fcimb.2013.00089

**Published:** 2013-11-27

**Authors:** Olivier Neyrolles, Elisabeth Mintz, Patrice Catty

**Affiliations:** ^1^Centre National de la Recherche Scientifique, Institut de Pharmacologie et de Biologie StructuraleToulouse, France; ^2^Institut de Pharmacologie et de Biologie Structurale, Université de Toulouse, Université Paul SabatierToulouse, France; ^3^Laboratoire de Chimie et Biologie des Métaux, Commissariat à l'Energie Atomique, Institut de Recherches en Technologies et Sciences pour le VivantGrenoble, France; ^4^Centre National de la Recherche Scientifique, UMR 5249Grenoble, France; ^5^UMR 5249, Université Grenoble-AlpesGrenoble, France

**Keywords:** *Mycobacterium tuberculosis*, macrophage, P-type ATPase, zinc, copper

Microbial killing inside macrophages and other phagocytes involves a variety of mechanisms, including, for instance, acidification of the phagocytosis vacuole—or phagosome—and the production of toxic oxygen and nitrogen radicals (Flannagan et al., 2009). In addition, the immune modulation of nutrients available for microbial development in infected cells and tissues is a re-emerging concept referred to as “nutritional immunity” (Weinberg, 1975). This concept mostly developed from knowledge of the intracellular microbial starvation mechanism involving phagosomal iron and manganese depletion through the metal transporter NRAMP (Hood and Skaar, 2012). Growing evidence suggests that immune defense against microorganisms also involves microbial killing by transition metals, such as zinc and copper, present in excess in the microbial environment, and a set of recent reports showed that several bacterial pathogens, such as the tuberculosis (TB) bacillus, *Mycobacterium tuberculosis*, require transition metal efflux and detoxification systems to thrive inside their host.

Zinc and copper play key functions in all biological systems. Bioavailable levels of zinc are sufficiently low that most microbes have evolved high affinity transport systems to capture this metal. Bacterial zinc transporters are usually ABC transporters consisting of a periplasmic-binding protein, a membrane permease, and an ATPase (Hantke, 2005). Proteins involved in zinc import in mycobacteria have yet to be discovered. Regarding copper, as in most bacterial species, uptake systems for this metal have not been identified in *M. tuberculosis*.

Metallobiology of zinc and copper in *M. tuberculosis* recently provided insights into novel host defense mechanisms against bacterial infection involving intoxication by these transition metals. To resist potential intoxication by metal ions (Nies, 1999; Mergeay et al., 2003; Silver and Phung, 2005), microbes express a range of metal efflux pumps and transporters belonging to three main families: heavy metal efflux members of the resistance–nodulation–cell division superfamily (HME-RND), the cation diffusion facilitators (CDF) family, and the P-type ATPase family (Nies, 2003). A set of recent studies strikingly reported that some of these efflux systems are required for microbial virulence in various bacterial species, including the TB bacillus, in order to resist newly described immune mechanisms relying on metal poisoning of microbes inside host cells. The *M. tuberculosis* genome (Cole et al., 1998; Nies, 2003) contains no member of the HME-RND family and only one putative CDF transporter (Rv2025c). In addition, it contains no member of the recently discovered MntX family involved in Mn^2+^ efflux (Veyrier et al., 2011) and no close homolog of ZntB, a member of the CorA family shown to mediate Zn^2+^ efflux in *Salmonella* (Worlock and Smith, 2002). However, the *M. tuberculosis* genome codes for the striking amount of 12 P-type ATPases, whose substrate specificity is still partially unknown and that might, at least for some of them, result from gene duplication and gene loss from common gene ancestors (Botella et al., 2012). In addition, mycobacteria possess a Cu^+^-binding metallothionein, MymT (Gold et al., 2008) that is part of a copper-regulated gene cluster, or regulon, regulated by the transcriptional repressor RicR (Festa et al., 2011), and that is involved in resistance to copper toxicity. MymT binds up to six Cu^+^ ions and may contribute to resistance to copper overload by either or both chelating copper inside the bacterial cytoplasm, or/and extruding copper through a yet to be identified transport system that might involve the LpqS and Rv2963 proteins (Festa et al., 2011).

Inference on selectivity of P-type ATPases for various metal ion species can be drawn from similarity to known transporters and from the presence of conserved metal-binding motifs. From a general viewpoint, metal specificity of a protein mostly follows the HSAB rule (Pearson, 1963). Briefly, hard acids like Na^+^, K^+^, Ca^2+^, or Mn^2+^ bind strongly to hard bases like carboxyl or hydroxyl groups whereas soft acids like Cu^+^ or Cd^2+^ prefer coordination with soft bases like thiol groups. This rule applies for metals transporters and is particularly well-illustrated with the ion selectivity of P-ATPases. Ion transport mechanism by P-ATPases consists in coupling between ATP hydrolysis occurring in the cytoplasmic domain of the transporter, and ion motion from one side to the other side of the bilayer, through the transmembrane (TM) domain of the transporter. This mechanism is very well-conserved throughout evolution. Ion selectivity of P-ATPases follows the HSAB rule and is determined by conserved amino acids, located at conserved positions in the TM domain of the transporter. For instance, Ca^2+^-ATPase binds Ca^2+^ thanks to Asp, Glu, Thr, and Asn residues located in TM helices 4, 5, 6, and 8. The same residues are found in the K^+^- and Na^+^-binding sites of the Na^+^/K^+^-ATPase (Bublitz et al., 2010). Metal transporting P_IB_-ATPases have been classified into five subfamilies on the basis of sequence homology (Arguello, 2003). Interestingly, this study reveals that each subfamily possesses conserved amino acids in TM helices 6, 7, and 8, likely to be involved in metal coordination, and these data, collected from *in silico* analysis of 249 sequences, corroborate biochemical data obtained on P_IB_-ATPases (Supplementary Table 1).

**Figure 1:**
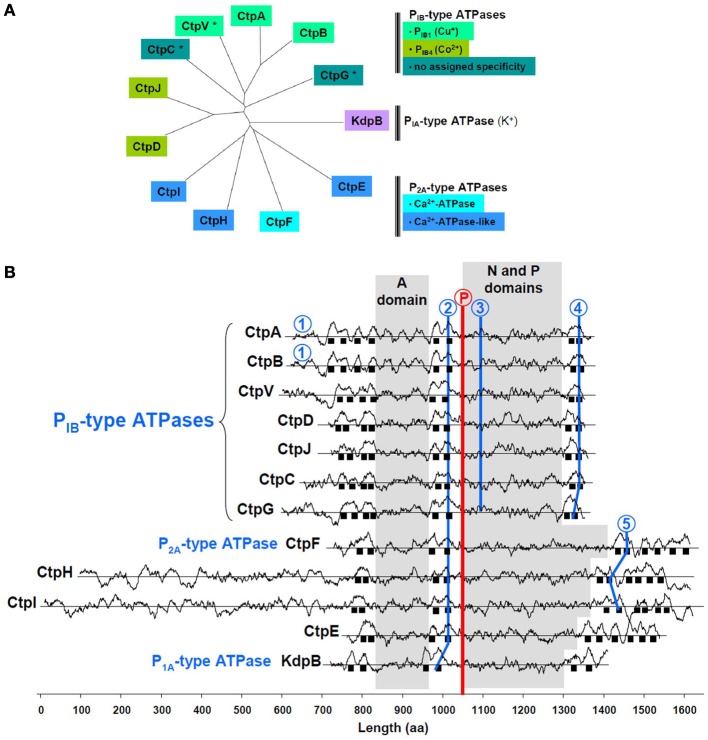
**Main feature of *M. tuberculosis* P-type ATPases. (A)** Sequences were aligned with ClustalW and organized in an unrooted tree using TreeView; ^*^, indicates the presence of a gene that could encode a metallochaperone, upstream the P-type ATPase encoding gene. **(B)** Hydrophobicity profiles are shown for the 12 P-type *M. tuberculosis* ATPases. Sequences are aligned on the phosphorylation motif (encircled P in red); black squares represent membrane spanning helices. Ion-binding motifs in P_1B_-type ATPases: 1, Cys-X_2_-Cys; 2, (Cys/Ser/Ala)-Pro-Cys; 3, Ser-(Glu/Arg)-His-(Pro/Ser/Ala); 4, Met-X_2_-Ser-Ser, His-Glu-Gln-X-Thr. Ion-binding motifs in P_2A_-type ATPases: 2, Pro-Glu-Gly-(Leu/Met)-Pro; 5, Leu-Trp-X-Asn-X_3_-Asp. A domain: actuator domain; N domain: nucleotide-binding domain; P domain: phosphorylation motif. The method to identify transmembrane domains has been previously described (Kyte and Doolittle, 1982), and is accessible on the ExPASy website: http://web.expasy.org/protscale/. The analysis was performed using a 21-amino acid window.

One of the *M. tuberculosis* P-type ATPases is KdpB, the ubiquitous P_IA_-type K^+^-ATPase (Figure 1A). Among the others, CtpA-D, CtpG, CtpJ, and CtpV display features of soft metal P_IB_-type ATPases (Arguello, 2003), i.e., a 4/2/2 arrangement of membrane spanning helices and specific sequence signatures in TM6, TM8, and the (N-P) domain (Figure 1B). Some of these sequences coincide with those determined from multiple alignments and allow assigning metal specificity. CtpA, CtpB, and maybe CtpV, would belong to the P_IB1_-type subfamily of Cu^+^-ATPases, while CtpD and CtpJ would belong to the P_IB4_-type subfamily of Co^2+^-ATPases. Less conserved motifs are found in CtpC and CtpG, preventing any prediction to be made about their metal selectivity. CtpE, CtpF, CtpH, and CtpI all exhibit a Pro-Glu-Gly-Leu-(Pro/Val) motif in the membrane spanning helix located upstream the phosphorylation site. This motif is found in all Ca^2+^-ATPases where it participates to the calcium transport site. In *M. tuberculosis*, only CtpF looks like a canonical Ca^2+^-ATPase, with a 2/2/6 arrangement of membrane spanning helices and a conserved motif in TM6.

The recent findings that *M. tuberculosis* mutants inactivated in *ctpV* and *ctpC*, are highly sensitive to copper and zinc, respectively (Ward et al., 2010; Botella et al., 2011; Padilla-Benavides et al., 2013), strongly suggest these two P-type ATPases transport these metal ions, but such a suggestion is not a formal proof of the metal selectivity. Biochemical characterization of these transporters in recombinant biological systems and in reconstituted liposomal fractions will be required to understand their exact function. In this context, a striking feature of three P-type ATPase members in *M. tuberculosis*, namely CtpC, CtpG, and CtpV, is the presence of a putative metallochaperone-encoding gene, namely and respectively Rv3269, Rv1993c, and Rv0968, upstream of the P-type ATPase-encoding genes that may play a part in metal selectivity and transport mechanism of their cognate P-type ATPase, as recently demonstrated for a similar transport system in *Streptococcus pneumoniae* (Fu et al., 2013).

A role for P-type ATPase-mediated metal detoxification in *M. tuberculosis* has been recently suggested by several independent reports. In particular *M. tuberculosis* mutants inactivated in the P-type ATPase-encoding genes *ctpV* and *ctpC* were shown to be impaired in their ability to proliferate in model animals and/or host macrophages (Ward et al., 2010; Botella et al., 2011). These results suggesting that *M. tuberculosis* is facing copper intoxication *in vivo* during infection were further strengthened by another report where it was shown that the outer membrane channel protein Rv1698/MctB is also required for both copper detoxification *in vitro* and for full virulence *in vivo* in guinea pigs (Wolschendorf et al., 2011). It was thus proposed that copper accumulation inside the mycobacterial phagosome might account for the phenotype of the Δ*ctpV* and Δ*mctB* mutants *in vivo* (Botella et al., 2012; Rowland and Niederweis, 2012; Samanovic et al., 2012). Several mechanisms have been proposed to explain copper ion toxicity, and the exact mechanism(s) of copper toxicity in *M. tuberculosis* remain to be identified (Rowland and Niederweis, 2012).

Regarding CtpC, we reported that genetic inactivation of this P-type ATPase dramatically increases *M. tuberculosis* sensitivity to Zn^2+^, which strongly suggested CtpC might be involved in zinc efflux (Botella et al., 2011). However, a recent report suggested that CtpC may transport Mn^2+^ over Zn^2+^, and that the hypersensitivity of the *ctpC* mutant to zinc may be due to an increased sensitivity to oxidative stress following impaired Mn^2+^ loading of the Fe^2+^-, and maybe Mn^2+^-, cofactored superoxide dismutase (SOD) SodA, and possibly other detoxification systems (Padilla-Benavides et al., 2013). In line with this hypothesis, it was shown recently that P-type ATPase-mediated copper export is required for copper supply to periplasmic Cu,Zn-SOD and resistance to oxidative stress in *Salmonella enterica* (Osman et al., 2013). Whether copper and zinc export through CtpV, CtpC, and possibly other P-type ATPases contributes to activation of the periplasmic Cu,Zn-SOD SodC in *M. tuberculosis* remains to be evaluated. Inside macrophages, we showed that zinc accumulates within *E. coli*- or *M. tuberculosis*-containing phagosomes, and that bacterial strains impaired in resistance to zinc (i.e., a Δ*zntA* mutant in *E. coli* or a Δ*ctpC* mutant in *M. tuberculosis*) are impaired in intracellular survival. *In vivo* attenuation of the *M. tuberculosis* Δ*ctpC* mutant still has to be clearly established (Botella et al., 2011; Padilla-Benavides et al., 2013). The apparent discrepancy between our results suggesting that CtpC transports zinc (Botella et al., 2011) and the results reported by Padilla-Benavides and colleagues, suggesting that this P-ATPase transports manganese over zinc and other metal ions (Padilla-Benavides et al., 2013), might be explained by the fact that these authors did not include the putative CtpC metallochaperone Rv3269 in their *in vitro* systems. Rv3269 contains a clear putative zinc-binding motif (DLHDHDH) in its C-terminus end, which might confer zinc-specificity to CtpC; this remains to be evaluated. Finally, the mechanism(s) of zinc ion toxicity in *M. tuberculosis* have yet to be discovered, and may include inactivation of iron-sulfur clusters, and inhibition of manganese uptake through transport competition in the bacterial periplasm (McDevitt et al., 2011; Xu and Imlay, 2012).

In summary, it is clear that *M. tuberculosis* uses P-type ATPases, such as CtpC and CtpV, and other systems, such as the metallothionein MymT, to resist poisoning by metal ions, such as Zn^2+^ and Cu^+^, and thrive inside its host. The function of the other *M. tuberculosis* P-type ATPases, and their possible implication in mycobacterial virulence, remains to be understood. Equally important will be to understand the function of the putative metallochaperones associated to CtpC, CtpG, and CtpV, which may open novel venues for the development of new therapeutic strategies.
