# Supplementary material for: Zinc and copper toxicity in host defense against pathogens: Mycobacterium tuberculosis as a model example of an emerging paradigm
Source: Front Cell Infect Microbiol. 2013 Nov 27;3:89. doi: 10.3389/fcimb.2013.00089 (PMC3841717; doi:10.3389/fcimb.2013.00089)
Supplement: Supplementary file 1 [file DataSheet1.DOCX]

**Supplementary Table 1. Characteristics of transmembrane (TM) helix motifs in P_IB-1-5_ P-type ATPases**

| **P-Atpase subfamily** | **TM helix 6** | **TM helix 8** | **References^a^** |
| --- | --- | --- | --- |
| IB-1 | CPC | MxxSS | ([Hung *et al*., 1997](#_ENREF_6);[Payne and Gitlin, 1998](#_ENREF_9);[Bissig *et al*., 2001](#_ENREF_3);[Voskoboinik *et al*., 2001a](#_ENREF_11);[Voskoboinik *et al*., 2001b](#_ENREF_12);[Fan and Rosen, 2002](#_ENREF_5);[Lowe *et al*., 2004](#_ENREF_7)) |
| IB-2 | CPC | DxG | ([Okkeri and Haltia, 2006](#_ENREF_8);[Wu *et al*., 2006](#_ENREF_13);[Dutta *et al*., 2007](#_ENREF_4)) |
| IB-3 | CPH | MSxST | {Arguello, 2003 #48} ([Arguello et al., 2007](#_ENREF_2)) |
| IB-4 | SPC | HEGxT | ([Seigneurin-Berny *et al*., 2006](#_ENREF_10);[Zielazinski *et al.*, 2012](#_ENREF_14)) |
| IB-5 | TPCP | QExxD | ([Arguello, 2003](#_ENREF_1)) |
